# Assessment of Reasons for Ownership and Attitudes About Policies Among Firearm Owners With and Without Children

**DOI:** 10.1001/jamanetworkopen.2021.42995

**Published:** 2022-01-14

**Authors:** Grace F. Ye, Priyanka Thatipamala, Michael Siegel

**Affiliations:** 1 Department of Pediatric Emergency Medicine, Boston Medical Center, Boston, Massachusetts; 2 Department of Family Medicine, University of California, San Diego; 3 Department of Public Health and Community Medicine, Tufts University School of Medicine, Boston, Massachusetts

## Abstract

**Importance:**

Given the dangers that firearms in the home pose to children, it is critical to engage parents in effective firearm safety counseling. This requires a broader understanding of how the presence of children in the home is associated with motivations surrounding gun ownership.

**Objective:**

To examine the association of having children in the home and gun owners’ attitudes and beliefs.

**Design, Setting, and Participants:**

This cross-sectional survey study analyzed data from the National Lawful Use of Guns Survey conducted in 2019. A representative sample of 3698 adult gun owners nationwide were randomly invited to participate, with a 56.5% survey response rate. Survey responses were weighted to account for survey nonresponse and selection bias, and comparison groups were matched by age. Statistical analysis was performed in 2020.

**Main Outcomes and Measures:**

Reasons for gun ownership, symbolic meaning of guns, and attitudes toward gun policies.

**Results:**

Of the 2086 respondents, 383 (18.4%) had children in the home, 68.7% (95% CI, 66.4%-71.0%) were male, 8.2% (95% CI, 6.8%-9.7%) were Black, 76.3% (95% CI, 73.8%-78.6%) were White, 79.4% (95% CI, 77.5%-81.2%) were living in metropolitan areas, 51.3% (95% CI, 48.9%-53.8%) identified as Republican; 34.7% (95% CI, 32.6%-36.9%) were aged 60 years or older. Despite the majority of respondents feeling safe in their local communities (respondents with children: 93.4% [95% CI,: 89.3%-96.0%]; without children: 88.9% [95% CI, 87.0%- 90.6%]), 92.3% (95% CI, 87.0%-95.6%) of respondents with children stated the primary reason for gun ownership was to protect their family, compared with 68.6% (95% CI, 65.2%-71.8%) of respondents without children. On logistic regression analysis, having children in the home remained an independent factor associated with reasons for gun ownership. Gun owners with children were more likely than those without children to feel that guns make them feel more valuable to their family (23.5% [95% CI, 18.9%-28.8%] vs 17.0% [95% CI, 15.0%-19.2%]). Among those with children, 35.2% (95% CI, 30.0%-40.8%) believed gun laws should be more strict compared with 40.7% (95% CI, 38.1%- 43.3%) of those without children.

**Conclusions and Relevance:**

These findings suggest that acknowledging parental motivations for gun ownership is a pivotal component of educational efforts toward firearm injury prevention. These findings can guide clinicians to engage in effective individual counseling and community level efforts to reduce pediatric gun injuries.

## Key Points

**Question:**

Is there an association between having children in the home and attitudes held by gun owners?

**Findings:**

In this national survey study of 2086 adult gun owners, those with children in the home were more likely to own guns for defense and protection of their family and were less likely to favor gun violence–prevention policies.

**Meaning:**

Clinicians should acknowledge the motivations and beliefs surrounding gun ownership when engaging parents in effective firearm injury counseling.

## Teaser

This survey study examines the association of having children in the home and attitudes and beliefs about firearms among US gun owners.

## Introduction

Every year in the United States (US), more than 3000 children and teenagers are killed by firearms, one-sixth unintentionally.^1,2,3^ For every child or teen fatally shot, another 5 experience nonfatal gunshot wounds.^4,5,6^ Gun violence is the second leading cause of death among children (aged 1 to 19 years) in the US^1,2^ and results in more than 20 000 emergency department visits annually.^7^ This presents a uniquely American problem. Children in the US are 15 times more likely to die from gunfire than their peers in 31 other high-income countries combined.^6^

Keeping a gun in the home increases both the intentional and unintentional mortality risks of household members, particularly the risk of suicide among adolescents.^8,9,10^ Guns stored in the home are associated with a 3-fold increase in the risk of homicide and a 5-fold increase in the risk of suicide,^11^ with most unintentional deaths of children (89%) occurring while playing with a loaded gun in their parent’s absence.^12,13^ When children are fatally injured by guns, the location is most often a home, and the gun most often originates from the home.^14^ In 2019, firearms accounted for 45% of adolescent suicides, with studies showing that firearms in the home, regardless of whether they are kept unlocked or stored locked, are associated with a higher risk of completed adolescent suicide.^2,15,16,17^ The American Academy of Pediatrics issued a policy statement in 2012 stating that “the absence of guns from children’s homes and communities is the most reliable and effective measure to prevent firearm-related injuries in children and adolescents.”^18^ Nevertheless, approximately one-third of US households with children have at least 1 gun.^19^

Previous studies have shown that perceptions surrounding pediatric gun safety varies widely. Notably, there appears to be a substantial disconnect in understanding the dangers of having a gun in the home. One study showed that “the majority of gun-owning parents (53%) endorsed safe storage as the best firearm injury prevention strategy, while 61% of parents who do not own firearms endorse not owning guns as the best way to prevent pediatric firearm injuries.”^3^ Another study showed that less than 10% of parents who own a gun believe that the best way to prevent firearm injuries to children is to remove the gun from the home.^20^ When asked, gun owners assert firmly that firearms in the home make the home safer.^21^ In contrast, less than 1% of pediatric residents nationwide believe that children are safer with a gun in the home.^22^

Other studies have investigated the motivations behind owning a gun. Despite the association between keeping a gun in the home and the increased risk of injury in children, studies have found that 61% to 88% of parents cite personal safety or protection as a reason for ownership.^23,24^ One-fourth of women with children believed a firearm would prevent a family member from being hurt in the case of a break-in, and 58% believed a firearm could scare off a burglar.^25^ However, little is known about the association of having children in the home and gun owners’ beliefs.

Given the dangers that firearms in the home pose to children, it is critical to engage parents in effective firearm safety counseling. This requires a broader understanding of how the presence of children in the home influences motivations surrounding gun ownership. Traditionally, gun culture in the US has been closely intertwined with personal identity and political affiliations. This creates conflicting social norms surrounding gun ownership that have been largely polarized and politicized in the media. As a result, it has been historically challenging to engage gun owners who are a part of this deeply rooted gun culture.^26^ Thus, a critical component of successful educational efforts in firearm injury prevention includes understanding the personal beliefs and perceptions that influence gun ownership. This study compares beliefs held by gun owners with children in their homes and those held by gun owners without children in their homes. We hypothesize that the presence of children in the home is associated with differing attitudes and beliefs held by gun owners, making them more likely to own guns for protection and perceive value to their family, and more likely to favor gun violence prevention policies.

## Methods

The National Lawful Use of Guns Survey (NLUGS) is a cross-sectional survey of 2086 gun owners across the US that was conducted in 2019 by Ipsos using the KnowledgePanel (KP).^27^ The KP is the largest probability sample-based national internet panel, consisting of 55 000 adult participants who were selected using representative sampling techniques. In order to obtain a goal sample size of 2000 with predicted survey response rates, 3698 KP members who reported owning a gun were randomly invited to participate, of whom 2086 completed the survey (56.5% survey response rate using American Association for Public Opinion Research Response Rate 1).^28^ The survey examined attitudes, practices, and lifestyles of gun owners nationwide. The NLUGS was judged to be exempt from review and informed consent by the Boston University Medical Center institutional review board because the investigators collected no data with personal identifying information. This study followed the American Association for Public Opinion Research (AAPOR) reporting guideline.

We used the data from the NLUGS data set to divide respondents into 2 groups: those who have children in the home (with children) and those who do not have children in the home (without children) (Figure). Variables examined in this study included (1) respondent characteristics (age, sex, race and ethnicity, education, marital status, annual income, employment status, urban status, political party and ideologies); (2) reasons for gun ownership (protection, value, and safety); (3) upbringing and symbolic meaning of guns; and (4) attitudes toward gun policies. Race and ethnicity data were self-reported, and the categories given as options were Hispanic, non-Hispanic Black, non-Hispanic White, and non-Hispanic other.

**Figure.**
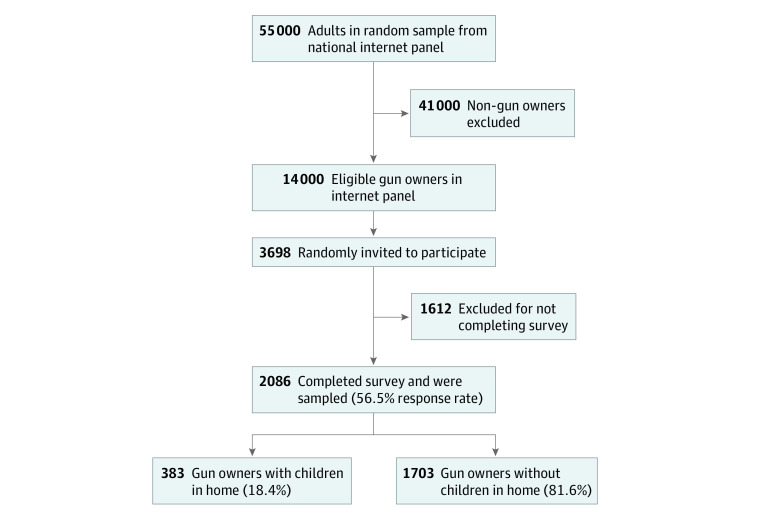
Study Population Derived From the National Lawful Use of Guns Survey (NLUGS)

### Statistical Analysis

Statistical analysis was performed in 2020 using STATA version 15.0 (StataCorp) and included descriptive statistics, χ^2^ tests of independence to compare categorical variables, *t* tests to compare continuous variables, and *z*-score tests to compare proportions of categorical response variables between subgroups of respondents. Logistic regression was performed for all binary variables that were statistically significant in the bivariate analysis. For all calculations, missing values for any variable were not included. Outcomes were described using weighted percentages and 95% CIs. The statistical significance threshold was set at *P* < .05. The NLUGS final survey results were weighted by Ipsos to account for survey nonresponse and selection bias with the intention of making the results representative of all US adult gun owners in 2019.^27^

## Results

### Respondent Characteristics

Of the 2086 people surveyed, 383 (18.4%) had children in the home, 68.7% (95% CI, 66.4%-71.0%) were male, 8.2% (95% CI, 6.8%-9.7%) were Black, 76.3% (95% CI, 73.8%-78.6%) were White, and 79.4% (95% CI, 77.5%-81.2%) were living in metropolitan areas; 34.7% (95% CI, 32.6%-36.9%) were aged 60 years or older. About half of the respondents identified as Republican (51.3% [95% CI, 48.9%-53.8%]) and described their political ideology as conservative (49.1% [95% CI, 46.7%-51.6%]). On average, respondents with children were younger than respondents without children, and a greater proportion had attended college, were married or living with a partner, working, and earning $100 000 per year or more (Table 1). When comparing the demographics of respondents and nonrespondents, respondents were more likely to be older, White, male individuals, but there were no significant differences in education, income, urbanicity, political party, or political affiliations.

**Table 1.** Characteristics of Adult Gun Owners in the US in 2019, Stratified by Presence of Children in the Home

Characteristics	Respondents, % (95% CI)		
	(N = 2086)	Without children (n = 1703)	With children (n = 383)
Sex			
Male	68.7 (66.4-71.0)	68.9 (66.3-71.3)	68.2 (62.7-73.3)
Female	31.3 (29.0-33.6)	31.1 (28.7-33.7)	31.8 (26.7-37.3)
Age, y			
<30	8.7 (7.2-10.5)	9.1 (7.4-11.1)	7.5 (4.8-11.5)
30-39	21.1 (18.9-23.5)	14.9 (12.7-17.3)	42.5 (37.0-48.3)
40-49	15.9 (14.2-17.8)	11.6 (9.9-13.6)	30.7 (25.9-35.9)
50-59	19.5 (17.8-21.4)	21.4 (19.3-23.5)	13.3 (10.4-16.8)
≥60	34.7 (32.6-36.9)	43.0 (40.5-45.6)	6.0 (4.3-8.4)
Race and ethnicity			
Hispanic	5.4 (4.3-6.9)	5.1 (3.8-6.8)	6.6 (4.0-10.7)
Non-Hispanic			
Black	8.2 (6.8-9.7)	8.3 (6.8-10.0)	7.7 (5.1-11.4)
White	76.3 (73.8-78.6)	76.7 (73.9-79.2)	75.0 (69.3-80.0)
Other^a^	10.1 (8.4-12.1)	9.9 (8.1-12.2)	10.6 (7.1-15.6)
Educational attainment			
Less than high school	6.6 (5.2-8.4)	6.8 (5.2-8.8)	6.0 (3.3-10.7)
High school	28.2 (26.0-30.5)	28.9 (26.5-31.5)	25.4 (20.7-30.9)
Some college	33.3 (31.1-35.6)	34.8 (32.3-37.4)	28.1 (23.5-33.2)
Bachelor’s or higher	31.9 (29.8-34.2)	29.5 (27.2-31.8)	40.5 (35.2-46.0)
Marital status			
Married or living with partner	73.4 (71.2-75.6)	69.2 (66.7-71.7)	87.9 (83.7-91.1)
Divorced, separated, widowed	15.4 (13.8-17.2)	17.9 (16.0-20.0)	6.9 (4.8-9.8)
Never married	11.1 (9.5-12.9)	12.8 (11.0-15.0)	5.2 (2.9-9.2)
Annual income, $			
<25 000	8.1 (6.8-9.6)	8.5 (7.1-10.1)	6.7 (4.3-10.4)
25 000-49 999	16.0 (14.3-17.9)	15.9 (14.1-18.0)	16.2 (12.3-21.0)
50 000-99 999	34.2 (31.9-36.5)	35.2 (32.6-37.7)	30.8 (26.0-36.1)
≥100 000	41.7 (39.4-44.2)	40.4 (37.8-43.1)	46.3 (40.7-51.9)
Employment status			
Working^b^	65.2 (62.9-67.4)	60.6 (58.0-63.1)	81.0 (76.1-85.0)
Retired	24.8 (22.9-26.7)	30.5 (28.3-32.9)	4.9 (3.3-7.4)
Not working^b^	10.0 (8.6-11.7)	8.9 (7.4-10.6)	14.1 (10.4-18.8)
Urban status			
Nonmetro	20.6 (18.8-22.5)	20.6 (18.6-22.8)	20.6 (16.5-25.3)
Metro	79.4 (77.5-81.2)	79.4 (77.2-81.4)	79.4 (74.7-83.5)
Political party			
Republican^c^	51.3 (48.9-53.8)	50.9 (48.2-53.6)	53.0 (47.3-58.5)
Independent^c^	22.8 (20.8-25.0)	23.1 (20.9-25.5)	21.8 (17.4-27.1)
Democrat^c^	25.8 (23.7-28.0)	26.0 (23.7-28.5)	25.2 (20.6-30.4)
Political ideology			
Conservative^d^	49.1 (46.7-51.6)	48.1 (45.5-50.8)	52.5 (46.9-58.1)
Moderate^d^	33.7 (31.4-36.1)	34.3 (31.8-37.0)	31.6 (26.5-37.2)
Liberal^d^	17.2 (15.4-19.1)	17.5 (15.6-19.6)	15.9 (12.1-20.6)

^a^ This option was listed as Other, non-Hispanic for respondents to choose.

^b^ Working includes working as a paid employee or self-employed; not working includes on temporary layoff, looking for work, disabled, or other.

^c^ Republican: strong Republican, not very strong Republican, or leans Republican; Independent: undecided, Independent, or other; Democrat: strong Democrat, not very strong Democrat, or leans Democrat.

^d^ Conservative: extremely conservative, conservative, or slightly conservative; moderate: moderate or middle of the road; liberal: extremely liberal, liberal, or slightly liberal.

### Reasons for Gun Ownership

Overall, most respondents stated that their primary reason for gun ownership was defense (respondents with children: 60.5% [95% CI, 55.0%-65.7%] vs without children: 59.0% [95% CI, 56.4%-61.6%]). Of those who answered defense, when asked more specifically in defense of whom; to protect their family, their community, or themselves, respondents with children were significantly more likely to answer to protect their family (92.3% [95% CI, 87.0%-95.6%]) compared with those without children (68.6% [95% CI, 65.2%-71.8%]) (Table 2). On logistic regression analysis, children in the home remained an independent factor associated with reasons for gun ownership, with an odds ratio (OR) of 1.42 (95% CI, 1.06-1.91).

**Table 2.** Reasons for Gun Ownership Among Adult Gun Owners in the US in 2019, Stratified by Presence of Children in the Home

Characteristics	Respondents, % (95% CI) (N = 2086)	
	Without children (n = 1703)	With children (n = 383)
Primary reason for owning a gun		
Defense^a^	59.0 (56.4-61.6)	60.5 (55.0-65.7)
Family	68.6 (65.2-71.8)	92.3 (87.0-95.6)
Self	31.1 (27.9-34.5)	7.0 (3.9-12.2)
Community	0.3 (0.1-0.9)	0.7 (0.1-4.7)
Recreation^a^	26.9 (24.7-29.3)	26.5 (22.1-31.4)
Rights/power^a^	8.0 (6.7-9.7)	7.5 (5.1-10.8)
Other^a^	6.0 (4.8-7.5)	5.5 (3.4-8.9)
Feels local community is safe^b^	88.9 (87.0-90.6)	93.4 (89.3-96.0)
Guns make me feel safe^c^	57.4 (54.7-60.0)	64.4 (58.9-69.5)
Mean response to: “Is gun violence a very big problem in your community?”, points (95% CI)^d^	2.37 (2.24-2.51)	2.04 (1.77-2.31)

^a^ Defense: to protect my family, to protect my community, or for my own protection; recreation: I enjoy hunting, I enjoy shooting for sport or competition, I like collecting them, or it is a tradition in my family; rights/ power: to exercise my constitutional rights, or they give me a feeling of power; other: to manage pests on my property, or I use them for my job.

^b^ Responded very safe or rather safe.

^c^ Responded strongly agree or agree.

^d^ On a scale of 0-10, with 0 being “no” and 10 being “yes.”

Regarding safety, respondents with children were more likely to state that guns make them feel safe (respondents with children: 64.4% [95% CI, 58.9%69.5%] vs without children: 57.4% [95% CI, 54.7%-60.0%]), and that gun violence was not a very big problem in their community (mean score among respondents with children: 2.04 points [95% CI, 1.77-2.31 points] vs without children: 2.36 points [95% CI, 2.24-2.51 points] on a scale of 0-10 points, 0 indicating no and 10 indicating yes as to whether gun violence is a big problem in the community). Interestingly, respondents with children were more likely to feel that their local community is safe (93.4% [95% CI, 89.3%-96.0%] vs 88.9% [95% CI, 87.0%-90.6%]). This difference persisted among those with children vs without children even when accounting for other demographics (eTable 1 in the Supplement).

### Upbringing and Symbolic Meaning of Guns

In terms of upbringing, most respondents grew up in households with guns (respondents without children: 67.7% [95% CI, 65.1%-70.2%]; with children: 67.7% [95% CI, 62.1%-72.9%]) and report that some to all of their family members own guns (respondents without children: 77.1% [95% CI, 74.8%-79.3%]; with children: 80.6% [95% CI, 75.7%-84.7%]). Despite this, most respondents disagreed with the notion that they own guns to uphold family tradition (Table 3).

**Table 3.** Upbringing, Identity, and Symbolic Meaning of Guns Among Adult Gun Owners in the US in 2019, Stratified by Presence of Children in the Home

Characteristics	Respondents, % (95% CI) (N = 2086)	
	Without children (n = 1703)	With children (n = 383)
Upbringing		
I grew up in a household with guns	67.7 (65.1-70.2)	67.7 (62.1-72.9)
My family members own guns^a^	77.1 (74.8-79.3)	80.6 (75.7-84.7)
I own firearms because it is a tradition in my family	32.0 (29.5-34.5)	36.8 (31.6-42.4)
Symbolic meaning of guns		
Make me feel confident	28.1 (25.7-30.7)	34.4 (29.2-40.0)
Make me feel more valuable to my family	17.0 (15.0-19.2)	23.5 (18.9-28.8)
Make me feel patriotic	22.0 (19.8-24.4)	30.3 (25.4-35.7)
Make me feel responsible	46.9 (44.2-49.6)	58.7 (53.1-64.1)
Make me feel in control of my fate	32.2 (29.7-34.8)	38.9 (33.5-44.5)
Make me feel respected	8.7 (7.2-10.5)	12.7 (9.3-17.3)
Make me feel empowered	14.5 (12.6-16.6)	20.4 (16.1-25.4)
Essential to my sense of freedom	62.5 (59.9-65.1)	64.8 (59.2-70.0)

^a^ Responded either all, most, or some.

Overall, respondents with children attributed a stronger symbolic meaning to guns (Table 3). Compared with the group without children, gun owners with children were more likely to agree that guns make them feel more confident, valuable to their family, patriotic, responsible, in control of their fate, and empowered. However, these differences were not seen between the children vs no-children groups when controlling for the remainder of the demographics. Respondents aged at least 50 years and those who identified as a Republican/Independent or with a conservative political ideology were more likely to attribute symbolic meaning to guns even when controlling for other confounders (eTable 2 in the Supplement).

### Attitudes Toward Gun Policies

Attitudes toward various gun policies are shown in Table 4. Overall, the majority of respondents supported universal background checks (respondents without children: 75.7% [95% CI, 73.3%-77.9%); with children: 72.6% [95% CI, 67.3%-77.3%]). Respondents with children were more likely to demonstrate opposition to gun laws. They were less likely to support stricter gun laws (respondents with children: 35.2% [95% CI, 30.0%-40.8%] vs without children: 40.7% [95% CI, 38.1%-43.3%]), and more likely to believe that the National Rifle Association (NRA) had too little influence over gun laws (with children: 24.2% [95% CI, 19.7%-29.4%] vs without children: 16.3% [95% CI, 14.3%18.4%]). Additionally, respondents with children were overall less likely to support policies aimed to limit the ability of people who are generally deemed to be at high risk of violence from obtaining guns. This includes preventing the mentally ill from purchasing guns, prohibiting a person convicted of a series of serious crimes as a juvenile from having a gun for 10 years, or barring gun purchases by people convicted of drunk and disorderly conduct or deemed to be a risk to themselves or others. The group with children were also less likely to oppose policies restricting guns with high violence potential (eg, banning high-capacity magazines for firearms, and banning military style semiautomatic assault weapons). Respondents with children were less likely to support people under 21 years of age from owning a gun (34.2% [95% CI, 29.1%-39.7%] vs 41.0% [95% CI, 38.3%-43.7%]), and this association remained when controlling for other factors. Only 37% (95% CI, 35.3%-40.6%) of respondents were in support of a law that requires a person lock up guns in their home when not in use, with no significant difference between those with and those without children. In terms of policies related to guns in schools, respondents with children were less likely to support policies restricting the carrying of concealed guns in elementary schools (33.5% [95% CI, 28.4%-39.0%] vs 42.8% [95% CI, 40.1%-45.4%]), or on college campuses (27.0% [95% CI, 22.3%-32.4%] vs 38.1% [95% CI, 35.5%-40.7%]).

**Table 4.** Attitudes Regarding Various Gun Laws and Policies Among Adult Gun Owners in the US in 2019, Stratified by Presence of Children in the Home

Characteristics	Respondents, % (95% CI) (N = 2086)	
	Without children (n = 1703)	With children (n = 383)
Believe gun laws should be		
More strict	40.7 (38.1-43.3)	35.2 (30.0-40.8)
Are about right	46.7 (44.0-49.4)	47.7 (42.2-53.3)
Less strict	12.7 (11.0-14.6)	17.1 (13.2-21.8)
Believe the influence of the NRA is		
Too much	34.5 (32.0-37.0)	29.4 (24.6-34.8)
Just right	49.3 (46.6-52.0)	46.4 (40.8-52.0)
Too little	16.3 (14.3-18.4)	24.2 (19.7-29.4)
Support for gun violence prevention policies^a^		
Prohibitor for mental illness	88.1 (86.1-89.9)	80.0 (75.3-84.1)
Prohibitor for no-fly list	73.5 (71.0-75.8)	68.1 (62.6-73.1)
Prohibitor for domestic violence restraining order	80.3 (78.0-82.4)	75.1 (70.0-79.7)
Prohibitor for ages <21 y	41.0 (38.3-43.7)	34.2 (29.1-39.7)
Prohibitor for drunk and disorderly conduct convictions	39.5 (36.9-42.2)	33.2 (28.2-38.7)
Universal background checks	75.7 (73.3-77.9)	72.6 (67.3-77.3)
Prohibitor for persons deemed risk to themselves or others	83.4 (81.2-85.3)	74.6 (69.4-79.2)
Restrict concealed gun carrying in elementary schools	42.8 (40.1-45.4)	33.5 (28.4-39.0)
Ban high-capacity ammunition magazines	40.5 (37.9-43.1)	28.6 (23.9-33.9)
Prohibitor for convictions of serious crimes as a juvenile	80.3 (78.0-82.4)	73.4 (68.2-78.0)
Require that all guns in home be locked when not in use	37.9 (35.3-40.6)	37.4 (32.1-43.0)
Restrict concealed gun carrying on college campuses	38.1 (35.5-40.7)	27.0 (22.3-32.4)
Ban assault weapons	44.5 (41.8-47.2)	30.0 (25.1-35.4)
Support for expanding gun rights^a^		
Allow concealed carry in more places	49.5 (46.9-52.2)	58.6 (52.9-64.0)
Allow teachers and officials to carry guns in K-12 schools	51.9 (49.2-54.6)	57.8 (52.2-63.3)

Abbreviation: NRA, National Rifles Association.

^a^ Respondents answered strongly support or support.

## Discussion

There are several important findings from our study. First, gun owners with children in the home share beliefs that guns keep their family safe and were more likely to state motivating reasons for ownership that align with protecting their family. They also believed that guns make them feel more valuable to their family. On logistic regression models, having children in the home remained an independent factor associated with reasons for gun ownership when controlling for other demographics. However, despite having this high concern for protecting their family, the overwhelming majority felt safe in their homes and believed that gun violence is not a very big problem in their communities. This discrepancy may present an opportunity for health care professionals to dispel blind acceptance of the perceived value of gun ownership, regardless of perceived threat. Studies have repeatedly shown that the vast majority of unintentional firearm-related injuries to children occur in the home.^3^ Health care professionals should acknowledge this gap between reality and perception, and be prepared to address the benefits vs risks that come with keeping a gun at home.

A second major finding was that, inconsistent with our hypothesis, gun owners with children were less likely to favor many gun violence prevention policies. First, those with children in their home were less likely to agree that gun laws needed to be more strict, and at the same time more likely to agree that the NRA had too little influence over gun laws. Second, a contrast between the groups is seen regarding support for banning guns with high violence potential. Third, respondents with children were noticeably more opposed to policy measures intended to limit gun ownership. Furthermore, the group with children showed stronger ties to the symbolic meaning of guns. This suggests a deeply engrained culture largely accepting of conventional NRA positions, which presents a potentially unique challenge to behavior modification. Many of these differences in belief did not persist between the groups with children vs without children when controlling for other demographics. However, we believe these findings can still be clinically meaningful when guiding clinicians in approaching effective pediatric firearm safety counseling with parents, as many of these factors are likely to have played a role in an individual’s decision to have children (eg, older respondents aged 40 years and older were more likely to share beliefs with the group with children). Additionally, in remembering that one of the goals of injury prevention counseling is to reach the appropriate audience (ie, acknowledging the dangers of firearm ownership in homes with children), the presence of children in these homes, despite the underlying reason, remains applicable.

Third, we found that respondents with children were not more likely to support a law requiring that a person lock up the guns in their home when not in use. Furthermore, on logistic regression, respondents with children in the home were less likely to support prohibiting those younger than 21 years of age from owning a gun. This is important because previous studies have shown that many parents have unrealistic perceptions of children’s capabilities and behavioral tendencies, and thus underestimate the danger of an unlocked firearm. Webster et al^29^ found that the majority of gun-owning parents believed that their child could tell the difference between a toy gun and real gun, and that instructing their children not to play with guns is sufficient to prevent accidents, with 23% believing that their child could be trusted with a loaded gun. Contrary to their parents’ reports, more than 65% of first and second graders know where their parents keep their firearms, and 36% admit handling the weapons.^13^ This implies that people own guns with the intention of protecting their family from harm, but are neither in support of policies nor willing to take the necessary measures to prevent the leading cause of firearm-related injury in children, which is accidental. These findings only further reinforce the importance of better communication.

In recent years, there have been substantial public health efforts and initiatives surrounding safe storage education. Despite this, studies have shown that having children in the home was not associated with higher rates of safe storage practices.^24^ It should also be noted that overall, the majority of respondents in our study oppose requiring a person to lock up their guns in their homes, suggesting that gun owners may be unreceptive toward these measures. If parents inherently believe that owning a gun makes their home safer, previous educational efforts centered around safe storage practices may not be the most effective approach toward counseling. Whether it be due to innately differing attitudes or simple knowledge misconception; without a receptive target audience, there can be no hope for collective behavioral changes. It is imperative that health care clinicians are able to demonstrate understanding from the perspective of the parent, and this continues to remain a critical component in implementing successful prevention strategies.

### Limitations

One limitation of our study is the potential for selection bias. The overall demographics of our survey sample were similar to those of gun owners in the 2015 National Firearms Survey,^30,31^ providing reassurance against considerable sampling bias. Our 57% survey completion rate was substantially greater than those typically seen in nonprobability, internet opt-in panels (around 2% to 16%).^32^ Additionally, comparison of respondent and nonrespondent demographics to assess the possibility of nonresponse bias revealed no difference in political party or ideology.

Although our random, diverse and large survey sample size enhances the generalizability of our study, we did not categorize the respondents based on region of the US State laws and regional political differences may produce variability in attitudes. Other factors that were not investigated in this study include mental health, substance abuse, family dynamics, religion, and socioeconomics, which could also contribute to shaping beliefs around gun ownership. It is notable that the age of children in the home was not examined, as parents may feel the risk of keeping a firearm in the home may differ with a toddler vs an adolescent. Although this is beyond the scope of this study, further studies should explore how parental perceptions of firearm-related risks are associated with childhood developmental stage. Lastly, respondents were not asked about safe storage practices at home or accessibility by children. It is possible that people would feel more inclined to own a gun with a child in the home if they did not believe the child could have access to it.

## Conclusions

Acknowledging the motivating factors and personal beliefs that are associated with one’s decision to own a gun is a pivotal component of educational efforts toward firearm injury prevention. Given the burden of pediatric firearm injuries, it is especially important to develop a deeper understanding of how parents’ motivations surrounding gun ownership are associated with having children in the home. Notably, gun owners continue to demonstrate strict attitudes opposing gun control measures that align strongly with a deeply engrained culture surrounding guns. This presents a unique challenge to health care clinicians when attempting to successfully engage gun owners. Clinicians should be cognizant of these discrepancies when targeting individual counseling and guiding community level efforts to reduce pediatric gun injuries.

## Notes

Supplement. **eTable 1.** Multivariable Logistic Regression of Reason for Gun Ownership and Symbolic Meaning of Guns **eTable 2.** Multivariable Logistic Regression of Attitudes Towards Gun Policies
