# Supplementary material for: Assessment of Reasons for Ownership and Attitudes About Policies Among Firearm Owners With and Without Children
Source: JAMA Netw Open. 2022 Jan 14;5(1):e2142995. doi: 10.1001/jamanetworkopen.2021.42995 (PMC8760615; doi:10.1001/jamanetworkopen.2021.42995)
Supplement: Supplement. — eTable 1. Multivariable Logistic Regression of Reason for Gun Ownership and Symbolic Meaning of Guns eTable 2. Multivariable Logistic Regression of Attitudes Towards Gun Policies [file jamanetwopen-e2142995-s001.pdf]

Supplemental Online Content

Ye GF, Thatipamala P, Siegel M. Assessment of reasons for ownership and attitudes about policies among firearm owners with and without children. *JAMA Netw Open.* 2022;5(1):e2142995. doi:10.1001/jamanetworkopen.2021.42995

eTable 1. Multivariable Logistic Regression of Reason for Gun Ownership and Symbolic Meaning of Guns

eTable 2. Multivariable Logistic Regression of Attitudes towards Gun Policies

This supplemental material has been provided by the authors to give readers additional information about their work.

© 2022 Ye GF et al. *JAMA Network Open.*

eTable 1. Multivariable Logistic Regression of Reason for Gun Ownership and Symbolic Meaning of Guns

	Own guns to protect family	Feels that local community is unsafe	Make me feel safe	Make me feel confident	Make me feel more valuable to my family
	N = 2072	*N = 2067*	*N = 2067*	*N = 2058*	*N = 2061*
**Variable**	**OR (95% CI)**	**OR (95% CI)**	**OR (95% CI)**	**OR (95% CI)**	**OR (95% CI)**
Children at Home					
Without Children	1 [Reference]	1 [Reference]	1 [Reference]	1 [Reference]	1 [Reference]
With Children	1.42 (1.06,1.91)	0.46 (0.24,0.87)	1.03 (0.76,1.40)	1.05 (0.76,1.44)	1.33 (0.90,1.94)
Sex					
Male	1 [Reference]	1 [Reference]	1 [Reference]	1 [Reference]	1 [Reference]
Female	0.65 (0.51,0.82)	1.3 (0.90,1.88)	1.17 (0.92,1.48)	1.19 (0.92,1.52)	0.74 (0.53,1.03)
Age, y					
<30	1 [Reference]	1 [Reference]	1 [Reference]	1 [Reference]	1 [Reference]
30 - 39	1.22 (0.73,2.05)	2.03 (0.93,4.45)	0.87 (0.51,1.50)	0.73 (0.44,1.21)	0.75 (0.43,1.33)
40 - 49	1.08 (0.64,1.81)	1.36 (0.61,3.03)	0.53 (0.31,0.91)	0.56 (0.34,0.94)	0.58 (0.31,1.09)
50 - 59	0.95 (0.57,1.57)	1.14 (0.53,2.47)	0.49 (0.29,0.83)	0.5 (0.30,0.81)	0.47 (0.27,0.83)
60+	0.61 (0.36,1.03)	1.17 (0.52,2.67)	0.32 (0.19,0.54)	0.29 (0.17,0.49)	0.42 (0.23,0.76)
Educational attainment					
Less Than High School	1 [Reference]	1 [Reference]	1 [Reference]	1 [Reference]	1 [Reference]
Some College or Higher	0.74 (0.58,0.94)	0.93 (0.61,1.42)	0.62 (0.47,0.80)	0.64 (0.48,0.85)	0.64 (0.45,0.89)
Race/Ethnicity					
White, non-Hispanic	1 [Reference]	1 [Reference]	1 [Reference]	1 [Reference]	1 [Reference]
Black, non-Hispanic	2.52 (1.63,3.89)	2.87 (1.63,5.04)	1.14 (0.74,1.76)	1.34 (0.81,2.22)	1.97 (1.13,3.44)
Other non-Hispanic	1.25 (0.82,1.90)	1.77 (0.94,3.34)	0.81 (0.52,1.25)	1.46 (0.93,2.30)	1.27 (0.74,2.18)
Hispanic	1.47 (0.87,2.51)	2.68 (1.18,6.07)	0.83 (0.47,1.47)	1.2 (0.66,2.18)	1.42 (0.70,2.89)
Marital status					
Married or living with partner	1 [Reference]	1 [Reference]	1 [Reference]	1 [Reference]	1 [Reference]
Divorced, separated, widowed	0.63 (0.47,0.84)	1.33 (0.86,2.06)	0.85 (0.63,1.13)	0.91 (0.65,1.26)	0.7 (0.47,1.04)
Never married	0.23 (0.14,0.36)	1.1 (0.57,2.14)	1.26 (0.84,1.89)	0.86 (0.55,1.33)	0.83 (0.48,1.42)
Annual income, $					
<25,000	1 [Reference]	1 [Reference]	1 [Reference]	1 [Reference]	1 [Reference]
25,000—49,999	1.13 (0.71,1.79)	0.61 (0.32,1.15)	0.79 (0.49,1.28)	0.64 (0.39,1.03)	0.85 (0.47,1.54)
50,000—99,999	1.05 (0.67,1.64)	0.46 (0.25,0.85)	0.92 (0.57,1.48)	0.71 (0.45,1.12)	1.11 (0.64,1.92)
≥100,000	0.76 (0.47,1.21)	0.28 (0.15,0.54)	0.78 (0.47,1.28)	0.57 (0.35,0.93)	0.78 (0.43,1.43)
Employment status					
Working[a]	1 [Reference]	1 [Reference]	1 [Reference]	1 [Reference]	1 [Reference]
Retired	1.38 (1.04,1.83)	0.98 (0.59,1.63)	1.02 (0.77,1.34)	1.07 (0.77,1.50)	1.26 (0.85,1.86)
Not working[a]	1.28 (0.85,1.90)	1.66 (0.94,2.91)	0.84 (0.55,1.29)	0.63 (0.41,0.98)	0.7 (0.41,1.18)
Urban status					
Non-metro	1 [Reference]	1 [Reference]	1 [Reference]	1 [Reference]	1 [Reference]
Metro	1.63 (1.26,2.10)	2.47 (1.44,4.22)	0.91 (0.70,1.16)	0.96 (0.72,1.26)	0.96 (0.70,1.32)
Political party					
Republican[b]	1 [Reference]	1 [Reference]	1 [Reference]	1 [Reference]	1 [Reference]
Independent[b]	1.01 (0.76,1.35)	1.61 (0.93,2.76)	0.78 (0.59,1.05)	0.69 (0.50,0.96)	0.9 (0.62,1.31)
Democrat[b]	0.77 (0.55,1.09)	1.32 (0.77,2.24)	0.45 (0.32,0.62)	0.51 (0.34,0.77)	0.56 (0.35,0.92)
Political ideology					
Conservative[c]	1 [Reference]	1 [Reference]	1 [Reference]	1 [Reference]	1 [Reference]
Moderate[c]	0.91 (0.70,1.18)	0.81 (0.50,1.31)	0.71 (0.54,0.92)	0.95 (0.71,1.27)	0.95 (0.68,1.34)
Liberal[c]	0.69 (0.47,1.01)	0.89 (0.51,1.55)	0.44 (0.30,0.63)	0.65 (0.42,1.01)	0.65 (0.37,1.14)

Abbreviations: N, number of observations; OR, odds ratio; 95% CI; 95% confidence interval; y, years

[a] Working: includes working as a paid employee or self employed; Not working: includes on temporary layoff, looking for work, disabled, or other

[b] Republican: strong Republican, not very strong Republican, or leans Republican; Independent: undecided, Independent, or other; Democrat: strong Democrat, not very strong Democrat, or leans Democrat

[c] Conservative: extremely Conservative, Conservative, or slightly Conservative; Moderate: Moderate or middle of the road; Liberal: extremely Liberal, Liberal, or slightly Liberal

© 2022 Ye GF et al. *JAMA Network Open.*

eTable 1. Multivariable Logistic Regression of Reason for Gun Ownership and Symbolic Meaning of Guns, continued

	**Make me feel patriotic**		**Make me feel responsible**		**Make me feel in control of my fate**		**Make me feel respected**		**Make me feel empowered**	
	N = 2060		***N = 2057***		***N = 2057***		***N = 2058***		***N = 2061***	
**Variable**	**OR (95% CI)**	**P value**	**OR (95% CI)**	**P value**	**OR (95% CI)**	**P value**	**OR (95% CI)**	**P value**	**OR (95% CI)**	**P value**
Children at Home										
Without Children	1 [Reference]		1 [Reference]		1 [Reference]		1 [Reference]		1 [Reference]	
With Children	1.05 (0.75,1.48)	0.77	1.27 (0.94,1.71)	0.12	1.04 (0.77,1.42)	0.79	1.15 (0.70,1.89)	0.57	1.11 (0.75,1.65)	0.59
Sex										
Male	1 [Reference]		1 [Reference]		1 [Reference]		1 [Reference]		1 [Reference]	
Female	0.77 (0.57,1.04)	0.09	0.87 (0.69,1.10)	0.23	1.11 (0.87,1.41)	0.42	1.01 (0.66,1.54)	0.97	1.01 (0.72,1.40)	0.97
Age, y										
<30	1 [Reference]		1 [Reference]		1 [Reference]		1 [Reference]		1 [Reference]	
30 - 39	0.48 (0.29,0.80)	< 0.001	0.88 (0.53,1.44)	0.6	0.81 (0.50,1.32)	0.4	0.76 (0.38,1.52)	0.44	0.83 (0.49,1.43)	0.51
40 - 49	0.33 (0.19,0.58)	< 0.001	0.76 (0.46,1.26)	0.28	0.7 (0.43,1.16)	0.16	0.61 (0.30,1.28)	0.19	0.44 (0.24,0.79)	0.01
50 - 59	0.25 (0.15,0.41)	< 0.001	0.55 (0.34,0.89)	0.01	0.53 (0.33,0.85)	0.01	0.36 (0.17,0.75)	0.01	0.36 (0.20,0.62)	< 0.001
60+	0.13 (0.074,0.23)	< 0.001	0.48 (0.30,0.78)	< 0.001	0.34 (0.21,0.56)	< 0.001	0.18 (0.081,0.41)	< 0.001	0.27 (0.15,0.50)	< 0.001
Educational attainment										
Less Than High School	1 [Reference]		1 [Reference]		1 [Reference]		1 [Reference]		1 [Reference]	
Some College or Higher	0.78 (0.57,1.06)	0.11	0.97 (0.76,1.25)	0.83	0.77 (0.59,1.01)	0.06	0.62 (0.39,0.98)	0.04	0.82 (0.57,1.17)	0.27
Race/Ethnicity										
White, non-Hispanic	1 [Reference]		1 [Reference]		1 [Reference]		1 [Reference]		1 [Reference]	
Black, non-Hispanic	0.99 (0.52,1.88)	0.98	0.75 (0.48,1.18)	0.21	1.01 (0.61,1.65)	0.99	1.38 (0.64,2.94)	0.41	1.33 (0.74,2.38)	0.35
Other non-Hispanic	0.97 (0.57,1.63)	0.9	1.06 (0.69,1.64)	0.79	1.48 (0.96,2.28)	0.08	1.53 (0.78,3.00)	0.22	1.07 (0.59,1.93)	0.82
Hispanic	1.03 (0.52,2.02)	0.93	1.22 (0.69,2.17)	0.5	1.05 (0.59,1.86)	0.87	0.85 (0.34,2.15)	0.73	0.86 (0.39,1.86)	0.7
Marital status										
Married or living with partner	1 [Reference]		1 [Reference]		1 [Reference]		1 [Reference]		1 [Reference]	
Divorced, separated, widowed	0.9 (0.62,1.30)	0.57	0.97 (0.72,1.29)	0.82	1.13 (0.83,1.53)	0.45	1.35 (0.80,2.30)	0.26	1.08 (0.68,1.69)	0.75
Never married	0.83 (0.49,1.41)	0.48	0.96 (0.65,1.44)	0.86	0.99 (0.65,1.51)	0.97	1.32 (0.66,2.65)	0.43	1.5 (0.90,2.52)	0.12
Annual income, $										
<25,000	1 [Reference]		1 [Reference]		1 [Reference]		1 [Reference]		1 [Reference]	
25,000—49,999	1.22 (0.69,2.15)	0.5	0.71 (0.45,1.14)	0.16	0.97 (0.58,1.60)	0.89	2.46 (1.05,5.77)	0.04	1.43 (0.74,2.78)	0.29
50,000—99,999	1.27 (0.74,2.17)	0.39	0.78 (0.50,1.21)	0.27	1.17 (0.73,1.86)	0.52	2.54 (1.12,5.77)	0.03	1.71 (0.91,3.22)	0.1
≥100,000	0.92 (0.52,1.62)	0.77	0.65 (0.41,1.04)	0.07	1.23 (0.75,2.02)	0.42	2.16 (0.89,5.26)	0.09	1.67 (0.84,3.31)	0.14
Employment status										
Working[a]	1 [Reference]		1 [Reference]		1 [Reference]		1 [Reference]		1 [Reference]	
Retired	1.13 (0.77,1.66)	0.53	0.89 (0.68,1.18)	0.43	1.25 (0.92,1.70)	0.16	1.54 (0.84,2.83)	0.17	0.85 (0.53,1.37)	0.5
Not working[a]	0.87 (0.55,1.40)	0.58	0.97 (0.66,1.42)	0.86	0.97 (0.64,1.46)	0.87	0.99 (0.52,1.88)	0.97	1.18 (0.72,1.92)	0.51
Urban status										
Non-metro	1 [Reference]		1 [Reference]		1 [Reference]		1 [Reference]		1 [Reference]	
Metro	1.04 (0.76,1.42)	0.79	0.94 (0.73,1.21)	0.63	0.93 (0.72,1.22)	0.61	0.96 (0.61,1.51)	0.85	1.04 (0.73,1.48)	0.84
Political party										
Republican[b]	1 [Reference]		1 [Reference]		1 [Reference]		1 [Reference]		1 [Reference]	
Independent[b]	0.53 (0.37,0.77)	< 0.001	0.79 (0.59,1.05)	0.1	1.01 (0.75,1.37)	0.93	0.53 (0.30,0.93)	0.03	0.78 (0.51,1.18)	0.24
Democrat[b]	0.42 (0.26,0.68)	< 0.001	0.58 (0.41,0.80)	< 0.001	0.71 (0.49,1.03)	0.07	0.64 (0.33,1.25)	0.19	0.72 (0.43,1.21)	0.21
Political ideology										
Conservative[c]	1 [Reference]		1 [Reference]		1 [Reference]		1 [Reference]		1 [Reference]	
Moderate[c]	0.66 (0.49,0.91)	0.01	0.78 (0.60,1.01)	0.06	0.73 (0.55,0.96)	0.02	0.96 (0.61,1.51)	0.86	1.07 (0.74,1.54)	0.73
Liberal[c]	0.27 (0.14,0.51)	< 0.001	0.55 (0.38,0.79)	< 0.001	0.46 (0.30,0.71)	< 0.001	0.65 (0.30,1.43)	0.29	0.87 (0.50,1.52)	0.62

Abbreviations: N, number of observations; OR, odds ratio; 95% CI; 95% confidence interval; y, years

[a] Working: includes working as a paid employee or self employed; Not working: includes on temporary layoff, looking for work, disabled, or other

[b] Republican: strong Republican, not very strong Republican, or leans Republican; Independent: undecided, Independent, or other; Democrat: strong Democrat, not very strong Democrat, or leans Democrat

[c] Conservative: extremely Conservative, Conservative, or slightly Conservative; Moderate: Moderate or middle of the road; Liberal: extremely Liberal, Liberal, or slightly Liberal

© 2022 Ye GF et al. *JAMA Network Open.*

eTable 2. Multivariable Logistic Regression of Attitudes towards Gun Policies

	Prohibitor for ages<21 years	Prohibitor for mental illness	Prohibitor for drunk and disorderly conduct convictions in last 10 years	Prohibitor for persons deemed risk to themselves or others	Restrict concealed gun carrying in elementary schools
	N = 2057	*N = 2062*	*N = 2049*	*N = 2053*	*N = 2054*
**Variable**	**OR (95% CI)**	**OR (95% CI)**	**OR (95% CI)**	**OR (95% CI)**	**OR (95% CI)**
Children at Home					
Without Children	1 [Reference]	1 [Reference]	1 [Reference]	1 [Reference]	1 [Reference]
With Children	0.72 (0.53,0.98)	0.8 (0.54,1.19)	0.78 (0.58,1.06)	0.77 (0.54,1.09)	0.94 (0.68,1.30)
Sex					
Male	1 [Reference]	1 [Reference]	1 [Reference]	1 [Reference]	1 [Reference]
Female	1.85 (1.45,2.35)	1.02 (0.71,1.45)	1.72 (1.36,2.17)	1.55 (1.10,2.18)	1 (0.78,1.27)
Age, y					
<30	1 [Reference]	1 [Reference]	1 [Reference]	1 [Reference]	1 [Reference]
30 - 39	0.71 (0.42,1.21)	0.98 (0.53,1.80)	0.82 (0.49,1.37)	1.31 (0.73,2.34)	1.14 (0.65,1.99)
40 - 49	0.98 (0.58,1.67)	1.5 (0.77,2.91)	0.85 (0.51,1.43)	1.55 (0.84,2.88)	1.31 (0.75,2.28)
50 - 59	0.89 (0.54,1.46)	2.11 (1.11,4.04)	0.85 (0.52,1.37)	2.53 (1.41,4.54)	1.78 (1.05,3.00)
60+	0.67 (0.40,1.11)	3.41 (1.71,6.83)	0.96 (0.58,1.57)	2.16 (1.17,3.98)	2.54 (1.49,4.32)
Educational attainment					
Less Than High School	1 [Reference]	1 [Reference]	1 [Reference]	1 [Reference]	1 [Reference]
Some College or Higher	0.82 (0.63,1.06)	0.89 (0.58,1.35)	0.97 (0.75,1.25)	1.01 (0.70,1.44)	1.1 (0.85,1.42)
Race/Ethnicity					
White, non-Hispanic	1 [Reference]	1 [Reference]	1 [Reference]	1 [Reference]	1 [Reference]
Black, non-Hispanic	2.56 (1.66,3.95)	0.87 (0.45,1.70)	1.1 (0.71,1.71)	0.6 (0.32,1.13)	1.27 (0.79,2.05)
Other non-Hispanic	1.78 (1.17,2.70)	1.12 (0.58,2.19)	1.59 (1.04,2.44)	0.74 (0.42,1.31)	0.77 (0.49,1.23)
Hispanic	1.4 (0.80,2.43)	1.18 (0.50,2.74)	1.27 (0.71,2.27)	0.75 (0.39,1.44)	0.85 (0.45,1.61)
Marital status					
Married or living with partner	1 [Reference]	1 [Reference]	1 [Reference]	1 [Reference]	1 [Reference]
Divorced, separated, widowed	1.14 (0.85,1.52)	0.74 (0.47,1.15)	0.82 (0.61,1.11)	0.9 (0.60,1.34)	1.15 (0.86,1.54)
Never married	0.94 (0.62,1.42)	0.88 (0.49,1.57)	0.9 (0.60,1.36)	0.87 (0.52,1.45)	0.87 (0.56,1.35)
Annual income, $					
<25,000	1 [Reference]	1 [Reference]	1 [Reference]	1 [Reference]	1 [Reference]
25,000—49,999	0.91 (0.57,1.44)	1.47 (0.79,2.74)	0.92 (0.57,1.48)	1.51 (0.85,2.68)	0.82 (0.49,1.38)
50,000—99,999	0.76 (0.49,1.19)	1.48 (0.81,2.69)	1.12 (0.71,1.75)	1.88 (1.10,3.23)	0.73 (0.45,1.20)
≥100,000	0.89 (0.56,1.42)	1.74 (0.91,3.32)	1.04 (0.64,1.67)	1.65 (0.93,2.90)	0.73 (0.44,1.21)
Employment status					
Working[a]	1 [Reference]	1 [Reference]	1 [Reference]	1 [Reference]	1 [Reference]
Retired	1.18 (0.89,1.57)	1.34 (0.82,2.17)	1.08 (0.81,1.43)	1.53 (1.04,2.26)	1.24 (0.94,1.65)
Not working[a]	1.01 (0.68,1.49)	0.72 (0.43,1.21)	1.11 (0.75,1.63)	1.08 (0.66,1.77)	1.06 (0.68,1.64)
Urban status					
Non-metro	1 [Reference]	1 [Reference]	1 [Reference]	1 [Reference]	1 [Reference]
Metro	1.66 (1.26,2.19)	0.98 (0.67,1.44)	1.35 (1.04,1.75)	1.26 (0.91,1.74)	1.33 (1.01,1.75)
Political party					
Republican[b]	1 [Reference]	1 [Reference]	1 [Reference]	1 [Reference]	1 [Reference]
Independent[b]	1.1 (0.81,1.49)	1.23 (0.78,1.94)	1.07 (0.79,1.44)	0.88 (0.60,1.27)	1.21 (0.90,1.64)
Democrat[b]	1.82 (1.29,2.55)	2.39 (1.31,4.35)	1.51 (1.08,2.10)	3.05 (1.76,5.28)	3.46 (2.44,4.90)
Political ideology					
Conservative[c]	1 [Reference]	1 [Reference]	1 [Reference]	1 [Reference]	1 [Reference]
Moderate[c]	1.2 (0.91,1.58)	1.19 (0.80,1.77)	1.18 (0.90,1.55)	1.97 (1.37,2.83)	1.8 (1.36,2.39)
Liberal[c]	1.16 (0.80,1.67)	0.7 (0.39,1.28)	1.19 (0.83,1.71)	1.6 (0.90,2.84)	2.48 (1.71,3.59)

Abbreviations: N, number of observations; OR, odds ratio; 95% CI; 95% confidence interval; y, years

[a] Working: includes working as a paid employee or self employed; Not working: includes on temporary layoff, looking for work, disabled, or other

[b] Republican: strong Republican, not very strong Republican, or leans Republican; Independent: undecided, Independent, or other; Democrat: strong Democrat, not very strong Democrat, or leans Democrat

[c] Conservative: extremely Conservative, Conservative, or slightly Conservative; Moderate: Moderate or middle of the road; Liberal: extremely Liberal, Liberal, or slightly Liberal

© 2022 Ye GF et al. *JAMA Network Open.*

eTable 2. Multivariable Logistic Regression of Attitudes towards Gun Policies, continued

	Ban high-capacity ammunition magazines	Prohibitor for convictions of serious crimes as a juvenile	Restrict concealed gun carrying on college campuses	Ban assault weapons	Allow concealed carry in more places
	N = 2056	*N = 2058*	*N = 2056*	*N = 2058*	*N = 2059*
**Variable**	**OR (95% CI)**	**OR (95% CI)**	**OR (95% CI)**	**OR (95% CI)**	**OR (95% CI)**
Children at Home					
Without Children	1 [Reference]	1 [Reference]	1 [Reference]	1 [Reference]	1 [Reference]
With Children	0.92 (0.66,1.29)	0.81 (0.58,1.14)	0.87 (0.62,1.22)	0.8 (0.56,1.14)	1.09 (0.80,1.48)
Sex					
Male	1 [Reference]	1 [Reference]	1 [Reference]	1 [Reference]	1 [Reference]
Female	1.64 (1.28,2.09)	1.24 (0.93,1.66)	0.99 (0.77,1.27)	1.82 (1.42,2.34)	0.79 (0.62,1.00)
Age, y					
<30	1 [Reference]	1 [Reference]	1 [Reference]	1 [Reference]	1 [Reference]
30 - 39	1.29 (0.73,2.30)	1.03 (0.56,1.90)	1.24 (0.68,2.26)	1.15 (0.65,2.05)	0.8 (0.47,1.35)
40 - 49	1.21 (0.67,2.16)	0.9 (0.49,1.65)	1.39 (0.77,2.48)	1.27 (0.71,2.25)	0.86 (0.51,1.47)
50 - 59	2.24 (1.31,3.82)	1.08 (0.60,1.93)	2.08 (1.20,3.63)	2.11 (1.24,3.59)	0.82 (0.49,1.36)
60+	3.43 (2.00,5.89)	1.48 (0.81,2.72)	2.54 (1.45,4.46)	3.07 (1.79,5.27)	0.41 (0.24,0.68)
Educational attainment					
Less Than High School	1 [Reference]	1 [Reference]	1 [Reference]	1 [Reference]	1 [Reference]
Some College or Higher	1.11 (0.85,1.43)	0.97 (0.70,1.35)	1.04 (0.79,1.36)	0.9 (0.68,1.19)	0.84 (0.64,1.11)
Race/Ethnicity					
White, non-Hispanic	1 [Reference]	1 [Reference]	1 [Reference]	1 [Reference]	1 [Reference]
Black, non-Hispanic	1.14 (0.71,1.85)	0.43 (0.27,0.70)	1.26 (0.77,2.06)	1.72 (1.00,2.95)	0.83 (0.50,1.35)
Other non-Hispanic	0.92 (0.58,1.45)	1.85 (0.95,3.61)	1.09 (0.67,1.76)	0.98 (0.64,1.52)	0.87 (0.55,1.37)
Hispanic	0.75 (0.41,1.38)	0.95 (0.50,1.79)	0.68 (0.36,1.27)	0.78 (0.41,1.51)	0.88 (0.52,1.48)
Marital status					
Married or living with partner	1 [Reference]	1 [Reference]	1 [Reference]	1 [Reference]	1 [Reference]
Divorced, separated, widowed	1.11 (0.83,1.49)	1 (0.70,1.42)	1.51 (1.12,2.04)	0.99 (0.74,1.33)	0.8 (0.60,1.08)
Never married	0.52 (0.33,0.82)	1.2 (0.71,2.04)	1.19 (0.74,1.90)	0.9 (0.56,1.46)	0.74 (0.49,1.12)
Annual income, $					
<25,000	1 [Reference]	1 [Reference]	1 [Reference]	1 [Reference]	1 [Reference]
25,000—49,999	1.52 (0.89,2.62)	1.11 (0.66,1.88)	1.09 (0.62,1.90)	1.05 (0.64,1.72)	0.88 (0.54,1.44)
50,000—99,999	1.49 (0.89,2.50)	1.28 (0.78,2.12)	1.2 (0.71,2.03)	0.97 (0.60,1.55)	0.87 (0.54,1.39)
≥100,000	1.3 (0.76,2.23)	1.35 (0.79,2.32)	1.45 (0.85,2.48)	1.05 (0.64,1.72)	0.68 (0.41,1.11)
Employment status					
Working[a]	1 [Reference]	1 [Reference]	1 [Reference]	1 [Reference]	1 [Reference]
Retired	1.45 (1.09,1.93)	1.2 (0.85,1.70)	1.38 (1.04,1.83)	1.41 (1.06,1.88)	0.91 (0.68,1.22)
Not working[a]	1.17 (0.76,1.81)	1.19 (0.74,1.90)	1.05 (0.67,1.65)	0.85 (0.55,1.31)	0.88 (0.58,1.34)
Urban status					
Non-metro	1 [Reference]	1 [Reference]	1 [Reference]	1 [Reference]	1 [Reference]
Metro	1.17 (0.89,1.53)	1.14 (0.85,1.53)	1.25 (0.93,1.66)	1.16 (0.89,1.52)	0.86 (0.67,1.11)
Political party					
Republican[b]	1 [Reference]	1 [Reference]	1 [Reference]	1 [Reference]	1 [Reference]
Independent[b]	1.01 (0.74,1.38)	1.08 (0.76,1.54)	1.13 (0.83,1.55)	1.13 (0.82,1.54)	0.83 (0.62,1.11)
Democrat[b]	3.2 (2.26,4.53)	1.71 (1.09,2.68)	3.36 (2.38,4.75)	4.17 (2.91,5.99)	0.36 (0.25,0.51)
Political ideology					
Conservative[c]	1 [Reference]	1 [Reference]	1 [Reference]	1 [Reference]	1 [Reference]
Moderate[c]	1.83 (1.37,2.43)	1.23 (0.90,1.68)	2.14 (1.60,2.86)	1.79 (1.34,2.40)	0.48 (0.37,0.63)
Liberal[c]	2.13 (1.46,3.10)	1.02 (0.62,1.66)	3.1 (2.12,4.52)	2.33 (1.57,3.45)	0.35 (0.24,0.51)

Abbreviations: N, number of observations; OR, odds ratio; 95% CI; 95% confidence interval; y, years

[a] Working: includes working as a paid employee or self employed; Not working: includes on temporary layoff, looking for work, disabled, or other

[b] Republican: strong Republican, not very strong Republican, or leans Republican; Independent: undecided, Independent, or other; Democrat: strong Democrat, not very strong Democrat, or leans Democrat

[c] Conservative: extremely Conservative, Conservative, or slightly Conservative; Moderate: Moderate or middle of the road; Liberal: extremely Liberal, Liberal, or slightly Liberal

© 2022 Ye GF et al. *JAMA Network Open.*
